# Overexpression of the *HDA15* Gene Confers Resistance to Salt Stress by the Induction of NCED3, an ABA Biosynthesis Enzyme

**DOI:** 10.3389/fpls.2021.640443

**Published:** 2021-04-30

**Authors:** Hai An Truong, Seokjin Lee, Cao Son Trịnh, Won Je Lee, Eui-Hwan Chung, Suk-Whan Hong, Hojoung Lee

**Affiliations:** ^1^Department of Plant Biotechnology, College of Life Sciences and Biotechnology, Korea University, Seoul, South Korea; ^2^Department of Molecular Biotechnology, College of Agriculture and Life Sciences, Bioenergy Research Center, Chonnam National University, Gwangju, South Korea

**Keywords:** *Arabidopsis*, *HDA15* gene, salinity, stress tolerance, histone modification, ABA accumulation

## Abstract

Salt stress constitutes a major form of abiotic stress in plants. Histone modification plays an important role in stress tolerance, with particular reference to salt stress resistance. In the current study, we found that HDA15 overexpression confers salt stress resistance to young seedling stages of transgenic plants. Furthermore, salt stress induces *HDA15* overexpression. Transcription levels of stress-responsive genes were increased in transgenic plants overexpressing *HDA15* (*HDA15 OE*). *NCED3*, an abscisic acid (ABA) biosynthetic gene, which is highly upregulated in *HDA15* transgenic plants, enhanced the accumulation of ABA, which promotes adaptation to salt stress. ABA homeostasis in *HDA15 OE* plants is maintained by the induction of CYP707As, which optimize endogenous ABA levels. Lastly, we found that the double-mutant *HDA15 OE/hy5 ko* plants are sensitive to salt stress, indicating that interaction between HDA15 and ELONGATED HYPOCOTYL 5 (HY5) is crucial to salt stress tolerance shown by *HDA15 OE* plants. Thus, our findings indicate that *HDA15* is crucial to salt stress tolerance in *Arabidopsis*.

## Introduction

Abiotic stresses damage more than 50% of average crop yields (Lisar et al., 2012; Eun et al., 2019). Unpredicted changes in the global climate expose plants to various environmental stresses at different times in their life cycle. Overpopulation leads to serious shortages of agricultural water. Therefore, plant water stress may pose a serious issue in the future (De Oliveira et al., 2013; Kim et al., 2019). Water stress may be associated with drought, salt stress, or temperature stress, and water shortages lead to various physiological phenomena (Pardo, 2010). Salt stress, which is considered a major form of abiotic stress in plants, inhibits plant growth due to excessive absorption of Na^+^ and Cl^−^, causing ion imbalances and physiological disorders (Gupta and Huang, 2014). Salt stress exerts several effects on plant growth. It induces osmotic stress, causing plant growth to decrease by reducing water influx, which decreases turgor pressure, enhances stomatal closure, and reduces photosynthetic activity. Since excess Na^+^ causes specific ionic toxicities, regulating the exclusion of Na^+^ as well as transportation of Na^+^ is paramount. The higher the concentration of Na^+^ and Cl^−^, the lower the absorption of other nutrients such as K^+^ and Ca^2+^, a process that leads to nutritional imbalance (Abassi et al., 2016). Moreover, reactive oxygen species (ROS) may be generated as the result of plant exposure to salt stress. Lipid peroxidation, protein degradation, and DNA mutations increase with increasing ROS (Abassi et al., 2016). Because plants are sessile, they cannot avoid salt stress and must continually adapt and evolve a variety of mechanisms to reduce the adverse effects of salt stress. Some of these mechanisms include the production of antioxidant enzymes to detoxify ROS, biosynthesis of compatible osmolytes, such as proline or glycine betaine, and accumulation of abscisic acid (ABA) (Gupta and Huang, 2014).

ABA is a crucial plant hormone that regulates the expression of stress-responsive genes under osmotic stress by functioning as an endogenous messenger that transmits stress signals. Osmotic stress activates ABA biosynthesis and inhibits ABA degradation (Fernando and Schroeder, 2016; Nam et al., 2019; Seo et al., 2019). Furthermore, it is known that the Salt Overly Sensitive (SOS) signaling pathway plays an important role in salt stress tolerance and ion homeostasis (Zhu, 2016). Histone modification, especially histone acetylation and deacetylation, plays a crucial role in the response to salt stress (Chen et al., 2010; Ueda et al., 2017; Zheng et al., 2019). ELONGATED HYPOCOTYL 5 (HY5), a master transcription factor (TF), belongs to the basic leucine zipper (bZIP) family (Gangappa and Botto, 2016). It inhibits hypocotyl elongation and lateral root growth but mainly regulates the light signaling pathway in *Arabidopsis* (Gangappa and Botto, 2016; Yang et al., 2018). Additionally, HY5 also plays a role in abiotic stress and the ABA signaling pathway. Germination of *hy5* mutant seeds is insensitive to the adverse effects of ABA, salt stress, and glucose, whereas *hy5* plants are less tolerant to salt stress and osmotic stress (Chen et al., 2008). Moreover, interaction between HY5 and RSM1 facilitates the binding of HY5 to the *ABI5* promoter, resulting in the upregulation of *ABI5* in the presence of salt stress or ABA (Chen et al., 2008). HY5 also induces the expression of cold-related genes and anthocyanin biosynthesis genes (Catalá et al., 2011). Thus, besides its light-dependent functions, HY5 broadly promotes the adaptation of *Arabidopsis* plants to abiotic stresses.

Histone acetylation and deacetylation in plants are two histone modifications that have been studied extensively. Histone acetylation involves the addition of acetyl groups to lysine residues within the histone tail at the N-terminus, a process that is catalyzed by histone acetyltransferases (HATs). However, during histone deacetylation, histone deacetyl transferases (HDACs) catalyze the removal of acetyl groups from lysine residues in the histone tail (Konsoula and Barile, 2012; Liu et al., 2016). Reportedly, histone acetylation loosens the DNA structure by neutralizing the positive charges on lysine residues, making the structure easily accessible to TF complexes, which bind the gene promoter and regulate gene expression (Henikoff, 2005; Shahbazian and Grunstein, 2007). There are 12 HATs that belong to four families: the GNAT family, MYST family, CBP family, and TAF_II_250 family (Pandey et al., 2002). Eighteen HDACs have been identified in *Arabidopsis*, and these are divided into three superfamilies as follows: (i) The Reduced Potassium Dependence 3/Histone Deacetylase 1 (RPD3/HDA1) superfamily contains 12 HDACs, which are further subdivided into three classes: Class I (HDA6, HDA7, HDA9, and HDA19), Class II (HDA5, HDA14, HDA15, and HDA18), and Class III (HDA2 and its two isoforms). Another RPD3/HDA1 superfamily contains HDA8, HDA10, and HDA17, which are still unclassified. (ii) Silent Information Regulator 2 superfamily (SIR2) contains SRT1 and SRT2. (iii) Histone Deacetylase 2 (HD2)-related protein family contains plant-specific HDACs and comprise four members (HD2A/HDT1, HD2B/HDT2, HD2C/HDT3, and HD2D/HDT4) (Liu X. et al., 2014). Both HATs and HDACs are associated with salt stress in *Arabidopsis*. GCN5, a member of the GNAT family, plays a role in salt stress tolerance by mediating cell wall-related genes in response to salt stress (Zheng et al., 2019). An *HDA6* mutant, *axe1-5*, and an *HDA6* RNAi line showed sensitivity to salt stress during seed germination (Chen et al., 2010). An *hda19* mutant in Col-0 background, *hda9*, as well as *AtHD2C* and *AtHD2D* overexpression lines are reportedly tolerant to salt stress (Sridha and Wu, 2006; Han et al., 2016; Zheng et al., 2016; Ueda et al., 2017). Conversely, quadruple mutants (*hda5/14/15/18*), an *hda19* mutant in Ws background, and an *hd2c* mutant were also reported to be sensitive to salt stress (Chen et al., 2010; Luo et al., 2012; Ueda et al., 2017).

A previous study found that HDA15 forms a complex with PIF1 and PIF3 to regulate the expression of light-responsive genes (Liu et al., 2013; Gu et al., 2017). Moreover, four Nuclear Factor-YC homologs in *Arabidopsis* redundantly interact with HDA15 to target hypocotyl elongation-related genes (Tang et al., 2017). Recently, HDA15 was found to positively regulate the suppression of *ROP* genes and ABA negative regulators by forming a complex with Myb96. Moreover, loss of function in *HDA15* was found to induce sensitivity to drought stress (Lee and Seo, 2019). Although *HDA15* is reportedly involved in salt stress in plants, the detailed mechanism underlying this process is not entirely known. In order to study the mechanisms underlying salt stress, plants overexpressing *HDA15* were generated and investigated to determine whether these plants were resistant than Col-0 plants under salt stress, anticipating that that this would lead to molecular mechanisms underlying HDA15 functions being revealed.

## Materials and Methods

### Plant Material, Growth Conditions, and Stress Treatments

Col-0 *Arabidopsis thaliana* (Col-0), *HDA15 OE* (transgenic, overexpression lines) were used in this study. The *hda15* mutant (SALK_004027), *hy5* mutant (*hy5-215*) was obtained from the *Arabidopsis* Biological Resource Center (ABRC). The seeds were surface sterilized and then incubated at 4°C in the dark for 3 days before germination. Half-strength MS media with 2% sucrose (pH = 5.7) were used as controls for plant growth. All seeds were grown at 23°C (16-h light/8-h dark) and 60% humidity for 3 days before being transferred to stressed media. For stress treatments, 4-day-old plants were subjected to 0, 175, or 200 mM NaCl stress treatment for 3 days. Plant phenotypes were captured photographically, survival rates were determined, and samples were collected for the chlorophyll assay. Forty plants were used per treatment (three replicates for each treatment), and the experiments were repeated three times independently. For vegetative salt stress treatment, after germination of seeds in 1/2 MS medium, they were raised for 10 days, and then seedlings of similar size were transferred to soil and allowed to grow for 3 more weeks before stress treatment. The plants were watered with 0 or 300 mM NaCl solutions for 8 days. Photographic images were obtained after 8 days from the initiation of stress, and the results were quantified by measuring the chlorophyll contents. Proline and MDA contents were also measured with the vegetative plant samples that were treated in the same manner.

### Germination Test

The seeds were sterilized and maintained at 4°C in the dark for 3 days. The sterilized seeds were then directly germinated on media containing 0, 50, 100 mM NaCl or 0, 0.1, 0.3, 0.5, 1 μM ABA for 8 days. Each condition contained three replicated plates. The results were recorded as percentages (%) of green cotyledons.

### DNA Construction and Transgenic Plant Generation

To generate *HDA15* overexpression lines, the cDNA of *HDA15* was amplified using RT-PCR with specific primers (Supplementary Table 1). The amplified fragments were then eluted and cloned into a TOPO vector (pCR8/GW/TOPO® TA Cloning Kit, Invitrogen). After sequence checking, *HDA15* was transferred to a pMDC32 vector under the control of a 35S CAMV promoter using gateway recombination. For plant transformation, Col-0 plants were used to implement a floral dipping method using *Agrobacterium tumefaciens* (*GV3101*). T_4_ homozygous plants overexpressing *HDA15* were utilized for all subsequent experiments.

To construct *HDA15pro::GUS*, we obtained ~1 kb of the *HDA15* promoter *via* PCR using a specific primer (Supplementary Table 1). The promoter was first cloned to a TOPO vector (pCR™8/GW/TOPO® TA Cloning Kit, Invitrogen) and then subcloned into a pMDC162 vector. *HDA15pro::GUS* was subsequently transformed into *Agrobacterium tumefaciens* (*GV3101*) by electroporation and then Col-0 plants *via* floral dipping.

### Lipid Peroxidation Assay

Three-week-old plants were challenged with 0 or 300 mM NaCl for 8 days, and the rosette leaves were collected and used. The assay was performed according to the previous study (Truong et al., 2017). Samples were homogenized using 0.1% (w/v) trichloroacetic acid (TCA) and centrifuged at 12,000 rpm, 4°C for 15 min. Thiobarbituric acid (0.5%) was diluted in 20% TCA and added to the supernatant, and the supernatant solution was incubated at 95°C for 25 min. The mixture was then placed on ice to terminate the reaction. Intensity of the developed color complex was measured at A532 and A600.

### Proline Content

Three-week-old plants were challenged with 0 or 300 mM NaCl for 10 days. Rosette leaves were sampled and used for this assay. The assay was performed according to a previous study (Truong et al., 2017). Samples were homogenized in 3% aqueous sulfosalicylic acid and the supernatant was collected after centrifugation at 12,000 rpm for 10 min. The mixture of supernatant, acidic ninhydrin, and glacial acetic acid was cultured at 100°C for 1 h, then transferred to ice to terminate the reaction. Toluene was used to extract the reaction mixture, and absorbance was measured at A520.

### qRT-PCR Analysis

For qRT-PCR analysis, 7-day-old plants were exposed to 150 mM NaCl or 1 μM ABA for 6 h. After that, samples were collected for RNA extraction and cDNA synthesis. Collected samples were used to isolate RNA, which was converted to cDNA following a previously described protocol (Jeong et al., 2018). Changes in the transcription levels of abiotic marker genes were detected *via* a qRT-PCR cycler (BioRad). Plant actin, *Actin2*, was used as the internal control. The experiment was repeated three times. The specific primers used in qRT-PCR analysis are listed (Supplementary Table 1).

### β-Glucuronidase Assay

T4 homozygous seedlings were used for the β-glucuronidase (GUS) assay. Seven-day-old seedlings were subjected to 150 mM NaCl for 6 and 24 h. The seedlings were then collected and used to either extract RNA for GUS quantification or observe intensive staining under the microscope. The experiments were repeated three times independently to maintain consistency.

### Abscisic Acid Content Measurement

For ABA content, 7-day-old seedlings were exposed to 150 mM NaCl for 24 h. Subsequently, 100-mg samples of seedlings were taken and ground in liquid nitrogen. Measurement of ABA content was performed according to a previous report (Liu N. et al., 2014) using a Phytodetek Elisa kit.

### Chlorophyll Assay

For the chlorophyll assay, samples from the abovementioned phenotype stress treatment were used to carry out chlorophyll determination as previously described (Truong et al., 2017). Briefly, ground samples were extracted using 80% acetone in the dark at room temperature (RT) for 45 min. The supernatant was collected following centrifugation at 3,000 rpm at 4°C for 15 min. Absorbance of chlorophyll was measured at A663 and A645. The experiments were repeated thrice independently to maintain consistency.

### Chromatin Immunoprecipitation Assay

Two-week-old plants were challenged using salt stress media containing 150 mM NaCl for 6 h. The samples were then subjected to fixation, sonication, and immunoprecipitation based on a previous protocol (Saleh et al., 2008). H3K14 and H4K16 antibodies were used in this assay. Purified DNA was analyzed *via* qRT-PCR with specifically designed primers at the 5′UTR and the exon.

### Data Analysis

Each experiment was repeated thrice to obtain reproducible results. SD value was calculated from three replicates of each treatment. Statistical Analysis System (SAS) was utilized to perform one-way ANOVA and Tukey's test (α = 0.05). Different letters (a, b, or c) within a treatment group indicate significant differences based on one-way ANOVA (*P* < 0.05).

## Results

### *HDA15* and Its Promoter Activity Is in Response to Salt Stress

**Figure 1:**
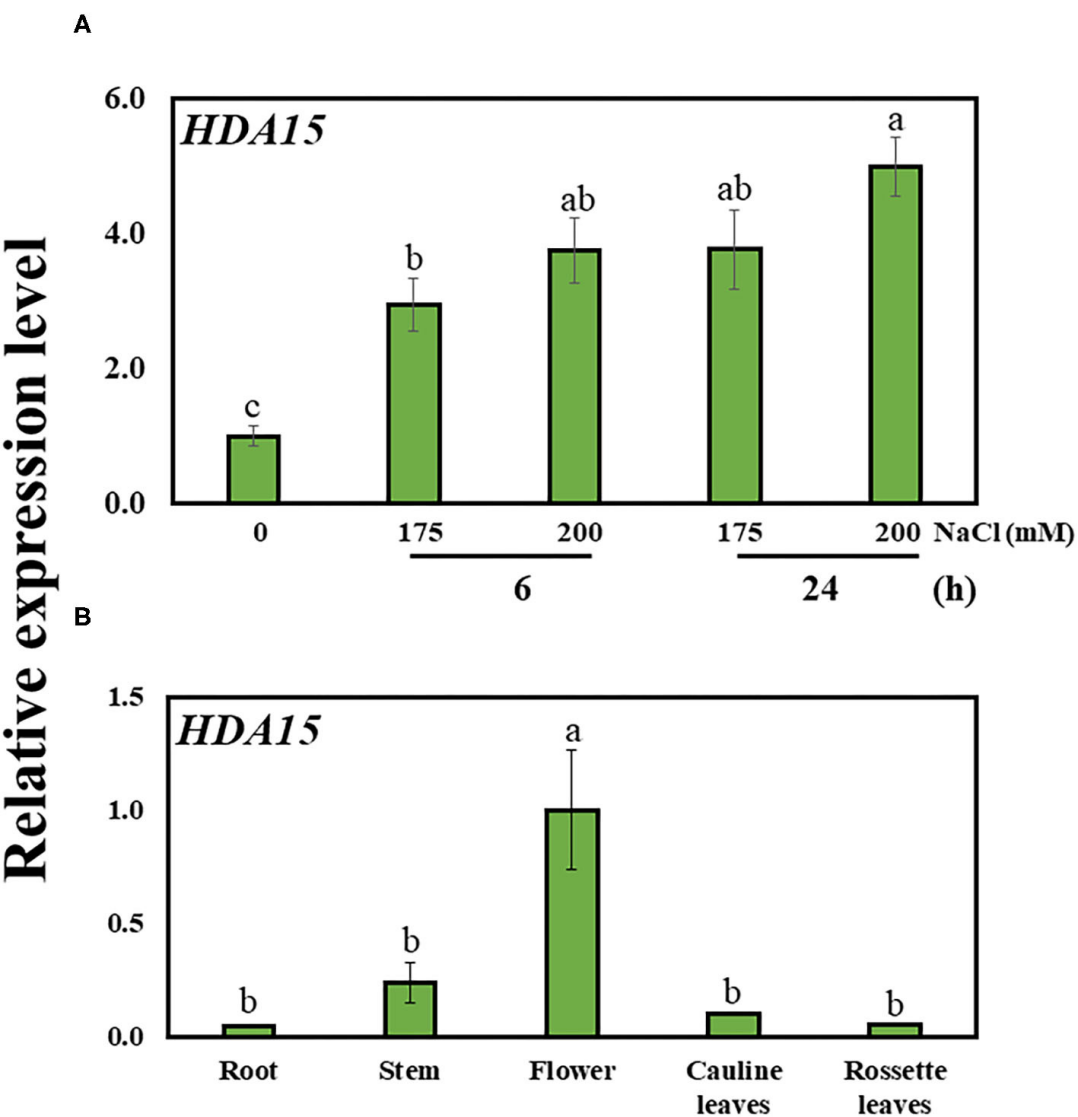
The expression of *HDA15* in wild-type plants. **(A)** *HDA15* expression in Col-0 plants in response to salt stress at 6 and 24 h. Seven-day-old Col-0 plants were exposed to 175 and 200 mM NaCl for 6 and 24 h followed by RNA extraction and cDNA synthesis for qRT-PCR. **(B)** The expression of *HDA15* in different tissues in Col-0 plants. Five-week-old Col-0 plants in soil pots were used to collect stems, flowers, cauline leaves, and rosette leaves. For root samples, 2-week-old Col-0 plants germinated in MS media were utilized. Error bars represent the standard deviation of three replicates. Different letters (a, b, or c) within a treatment group indicate significant differences based on one-way ANOVA (*P* < 0.05).

**Figure 2:**
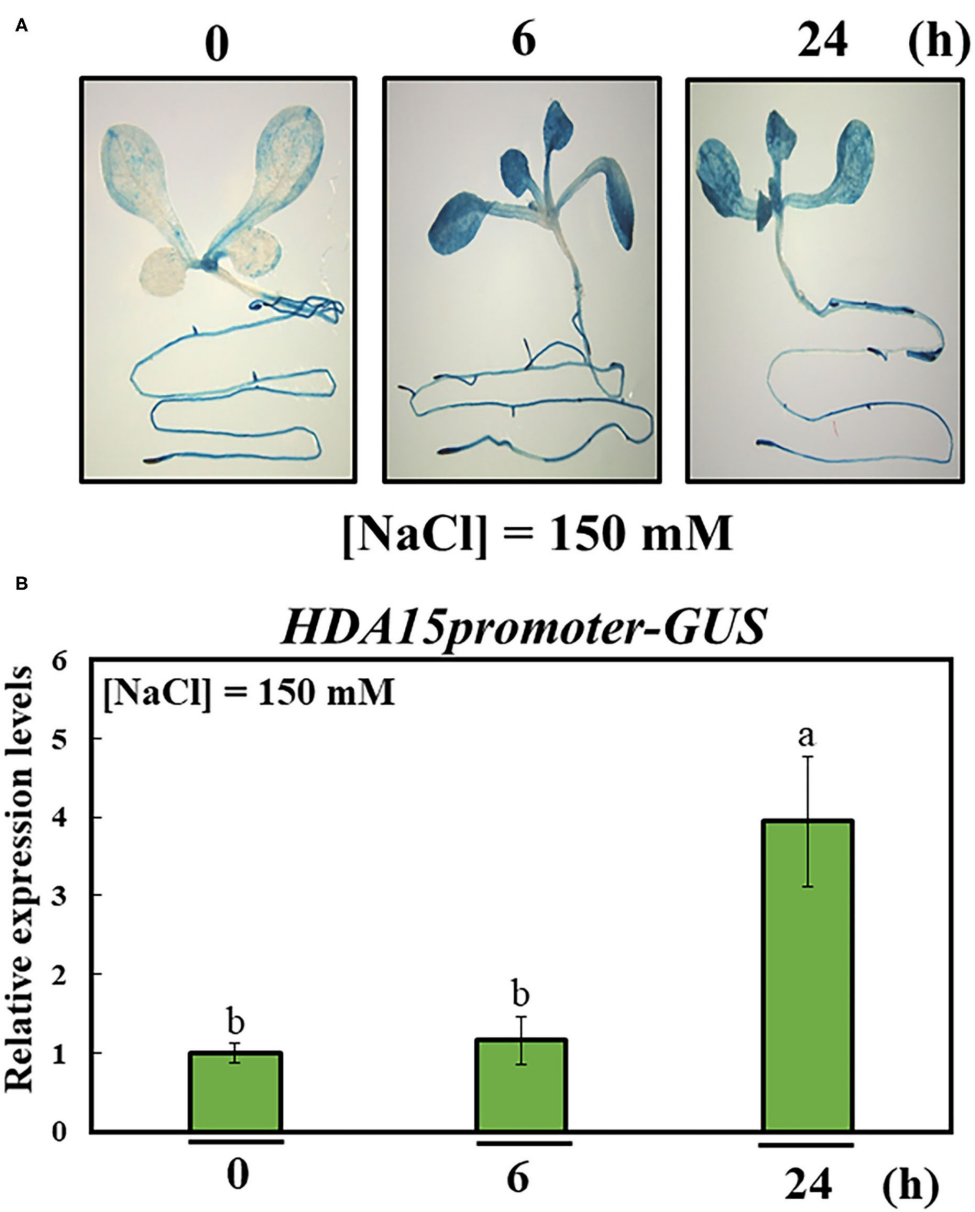
Activity of *HDA15* promoter in response to salt stress. The promoter of *HDA15* was cloned into a pMDC162 vector containing the *GUS* reporter gene *via* the Gateway system. Homozygous T_4_ seeds were used to perform β-glucuronidase (GUS) staining and quantification. **(A)** Visualization of the response of the *HDA15* promoter to salt stress. Seven-day-old plants were stained with GUS solution for 2 h to visualize promoter activity in response to 150 mM NaCl at 6 and 24 h. **(B)** GUS quantification of the *HDA15* promoter under salt stress. RNA was extracted from 7-day-old *HDA15pro::GUS* plants challenged by salt stress under the same conditions indicated above for GUS staining. The GUS primer was used to perform qRT-PCR. *Actin2* was used as an internal control. Error bars represent the standard deviation of three replicates. Different letters (a, b) within a treatment group indicate significant differences based on one-way ANOVA (*P* < 0.05).

In order to better understand the role of HDA15, we examined *HDA15* expression in Col-0 plants exposed to salt stress for 6 and 24 h. The transcript level of *HDA15* was increased at both 6 and 24 h, suggesting that *HDA15* rapidly reacted to salt stress (Figure 1A). *HDA15* expression was higher in flower tissues than in tissues such as roots, stems, cauline leaves, and rosette leaves (Figure 1B). Next, we questioned whether the increase seen in the transcription level of this gene was due to its promoter activity. Hence, homozygous T_4_ plants of the *HDA15pro::GUS* line were generated to visualize the promoter activities of *HDA15* in response to salt stress. Upon examining GUS expression in *HDA15pro::GUS* plants grown under normal conditions, we determined that the *GUS* reporter gene was evenly expressed in all tissues of young plants (Figure 2A). However, following the exposure of *HDA15pro::GUS* plants to salt stress at 150 mM NaCl for 6 and 24 h, *GUS* expression becomes much stronger throughout the plants (Figure 2). As evidenced by the quantitation results of *GUS* expression (Figure 2B), it appears that the activity of the *HDA15* promoter was significantly induced by salt stress.

### Overexpression of *HDA15* Enhanced the Tolerance of Transgenic Plants in Response to Salt Stress

**Figure 3:**
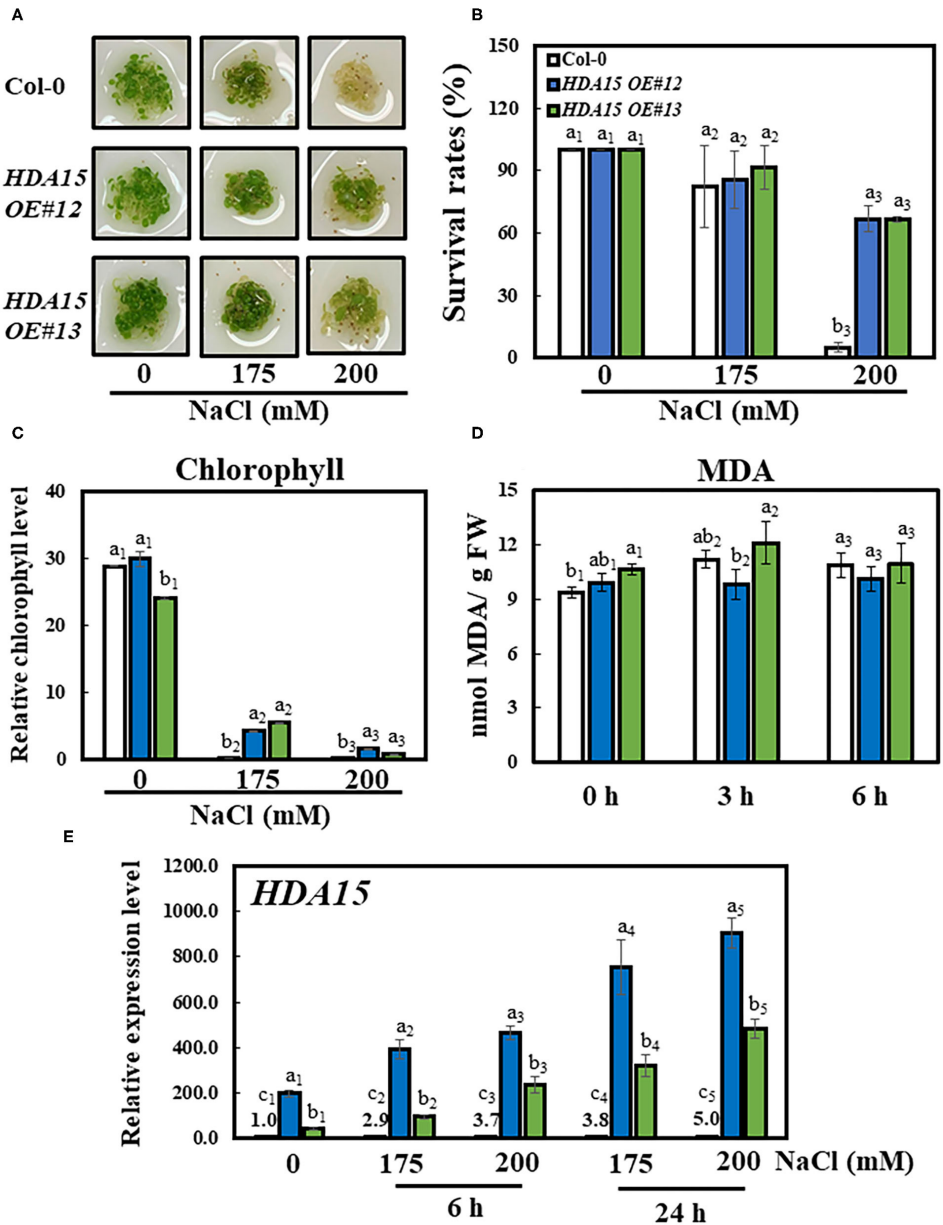
Growth performance of *HDA15* transgenic plants, survival rates, and chlorophyll content in response to salt stress. Four-day-old plants, which were germinated in normal MS media, were transferred to different NaCl concentrations as indicated. The experiment was repeated three times independently to obtain a consistent result. **(A)** Phenotypes of Col-0 and *HDA15 OE* transgenic plants following a 3-day exposure to salt stress. **(B)** The survival rates of Col-0 and *HDA15 OE* transgenic plants following a 3-day exposure to salt stress. **(C)** Chlorophyll levels of Col-0 and *HDA15 OE* transgenic plants following a 3-day exposure to salt stress. **(D)** The lipid peroxidation levels of Col-0 and *HDA15 OE* plants in response to salt stress. Seven-day-old plants, which were germinated in normal MS media, were transferred to 150 mM NaCl for 0, 3, and 6 h. **(E)** *HDA15* expression in Col-0 and *HDA15 OE* plants. Seven-day-old plants were exposed to 175 and 200 mM NaCl for 6 and 24 h, followed by RNA extraction and cDNA synthesis for qRT-PCR. *Actin2* was used as an internal control. Error bars represent the standard deviation of three replicates. Different letters (a, b, or c) within a treatment group indicate significant differences based on one-way ANOVA (*P* < 0.05).

To further investigate the function of *HDA15* under salt stress, we generated overexpression lines of *HDA15* (*HDA15 OE*) and tested the resulting phenotypes with 0, 175, and 200 mM NaCl treatment. No differences were observed between the growth performances of Col-0 and *HDA15 OE* plants under control conditions. However, *HDA15 OE* plants appeared greener under salt stress compared to Col-0 plants, most of which died after 3 days of being challenged by salt stress using 200 mM NaCl (Figure 3A). The results were also quantified as survival rates, which were equivalent to the proportion of plants with green cotyledons (Figure 3B). Exposure to 200 mM NaCl significantly decreased the survival rates of Col-0 plants (by ~95%) compared to those under normal conditions. However, exposure to 200 mM NaCl reduced the survival rates of *HDA15 OE* plants up to 35%, and this survival rate is higher than that of Col-0 under stress conditions (Figure 3B). In addition, chlorophyll contents were consistent with growth performance (Figure 3C). Although chlorophyll contents tended to decrease with salt stress in all types of plants, the decrease seen in *HDA15 OE* plants was less than that of Col-0.

Under control conditions, the transcript levels of *HDA15 OE#12* and *HDA15 OE#13* plants were increased more than 200 and 43 times, respectively, compared to those of the Col-0 plants. Moreover, salt stress increased the transcript levels of *HDA15* in transgenic plants by at least twice compared to those under normal conditions (Figure 3E). However, the *hda15 knock out* (*ko*) mutant did not show significant phenotypic alterations under salt stress when compared to Col-0 plants (Supplementary Figures 1A,B). In addition to investigating salt stress, we also examined the germination response of Col-0 and *HDA15 OE* plants in ABA media to determine whether *HDA15 OE* responds differently to ABA when compared with Col-0 (Supplementary Figures 1C,D). Under salt conditions, the percentage of germinated *HDA15 OE* plants was slightly less than that of Col-0 plants. The ratios of *HDA15 OE* plants with green cotyledons in response to exogenous ABA levels were slightly higher than those in Col-0 plants. Next, we measured lipid peroxidation, an indicator of oxidative stress. There was no significant difference between lipid peroxidation of Col-0 and *HDA15 OE* plants under salt stress (Figure 3D). Additionally, we tested the tolerance to salt stress of *HDA15 OE* plants in the vegetative stage (Supplementary Figure 2). Under salt stress, the leaves of Col-0 and *HDA15 OE* plants showed yellowing phenotype, indicating that salt stress is harmful to plant growth. As shown in Supplementary Figure 2A, *HDA15 OE* plants were less damaged than Col-0, which showed the same result in chlorophyll content measurement. According to these results, it is necessary to conduct more stress test experiments with various conditions to make a clear conclusion, but the *HDA15* effect can be considered to have more influence on young seedlings.

**Figure 4:**
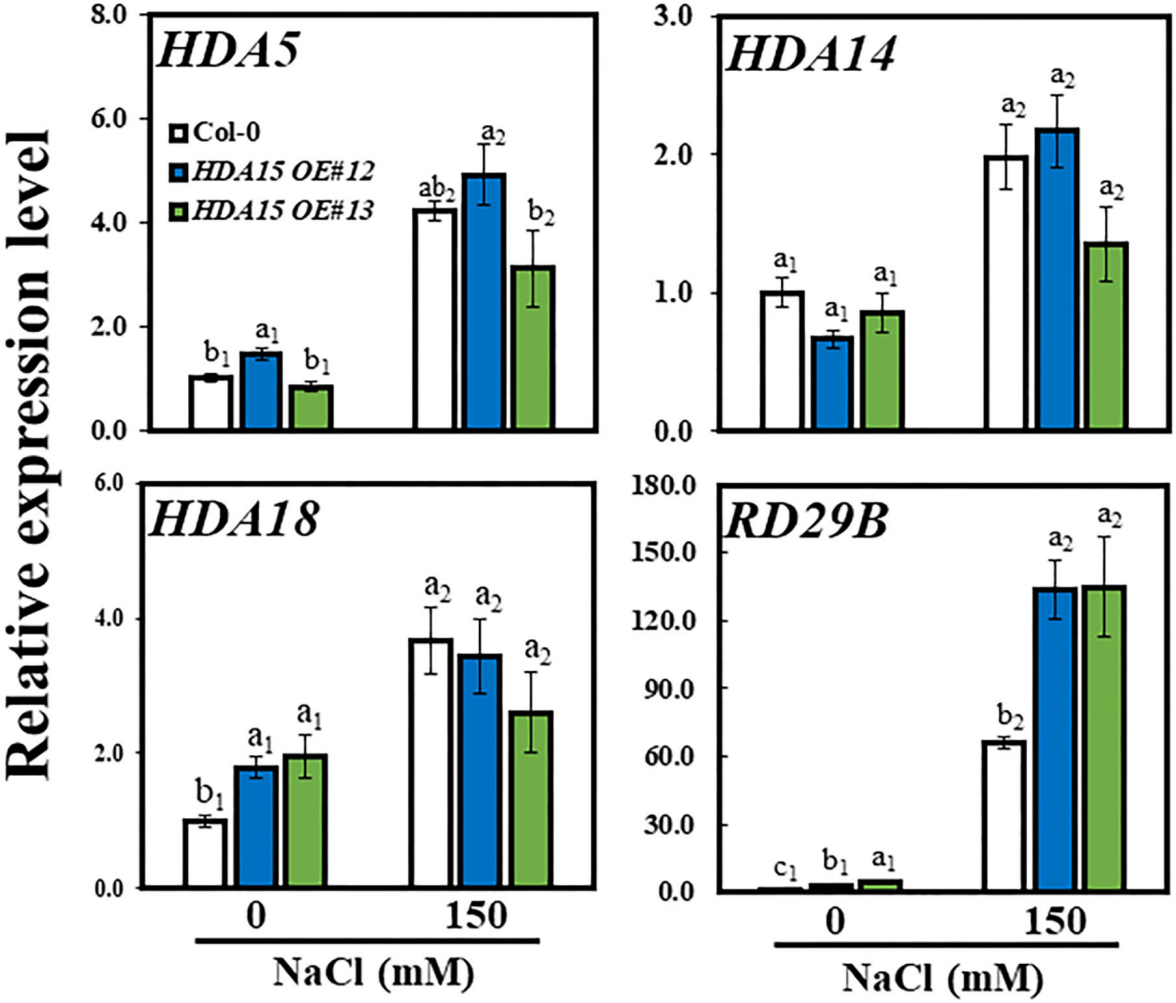
Expression levels of HDA15 homologs and a stress-related gene under salt stress. Seven-day-old plants were subjected to 150 mM NaCl for 6 h. RNA was then extracted, and cDNA was synthesized. Next, qRT-PCR was performed with primers for *HDA5, HDA14, HDA18*, and *RD29B* genes. *Actin2* was used as an internal control. The experiment was repeated three times independently. Error bars represent the standard deviation of three replicates. Different letters (a, b, or c) indicate significant differences based on one-way ANOVA (*P* < 0.05).

Enhanced tolerance of *HDA15 OE* plants in response to salt prompted us to examine the transcription level of *HDA15* and homologous genes including *HDA5/14/18* in response to salt stress (Figure 4). The transcript levels of all three homologs were increased in response to high salt in Col-0 plants, confirming that Class II HDACs are responsive to salt stress. However, the transcript levels of the three homologs in *HDA15 OE* plants were not different from those of Col-0 plants under stress, indicating that *HDA15* overexpression does not interfere with the expression of its homologs. To ensure our salt stress studies were properly conducted, we investigated the transcript level of an abiotic-stress marker gene, *RD29B*, a well-known gene that is induced by salt stress (Msanne et al., 2011). HDA15 transgenic plants showed increased transcript level of *RD29B* under salt stress (Figure 4), indicating that *HDA15 OE* plants regulate stress signaling more effectively than Col-0. Additionally, we detected a profile of downregulated genes in the *hda15 ko* mutant under normal conditions (RNA-Seq data; Zhao et al., 2019). These included some salt stress-responsive genes that downregulated transcript levels in the *hda15 ko* mutant, indicating that HDA15 serves as a positive regulator in the induction of some stress-responsive genes.

### Increased Transcript Levels of *NCED3*, an ABA Biosynthetic Gene, Enhance Salt Stress Tolerance of Plants *via* Enhanced Abscisic Acid Accumulation

**Figure 5:**
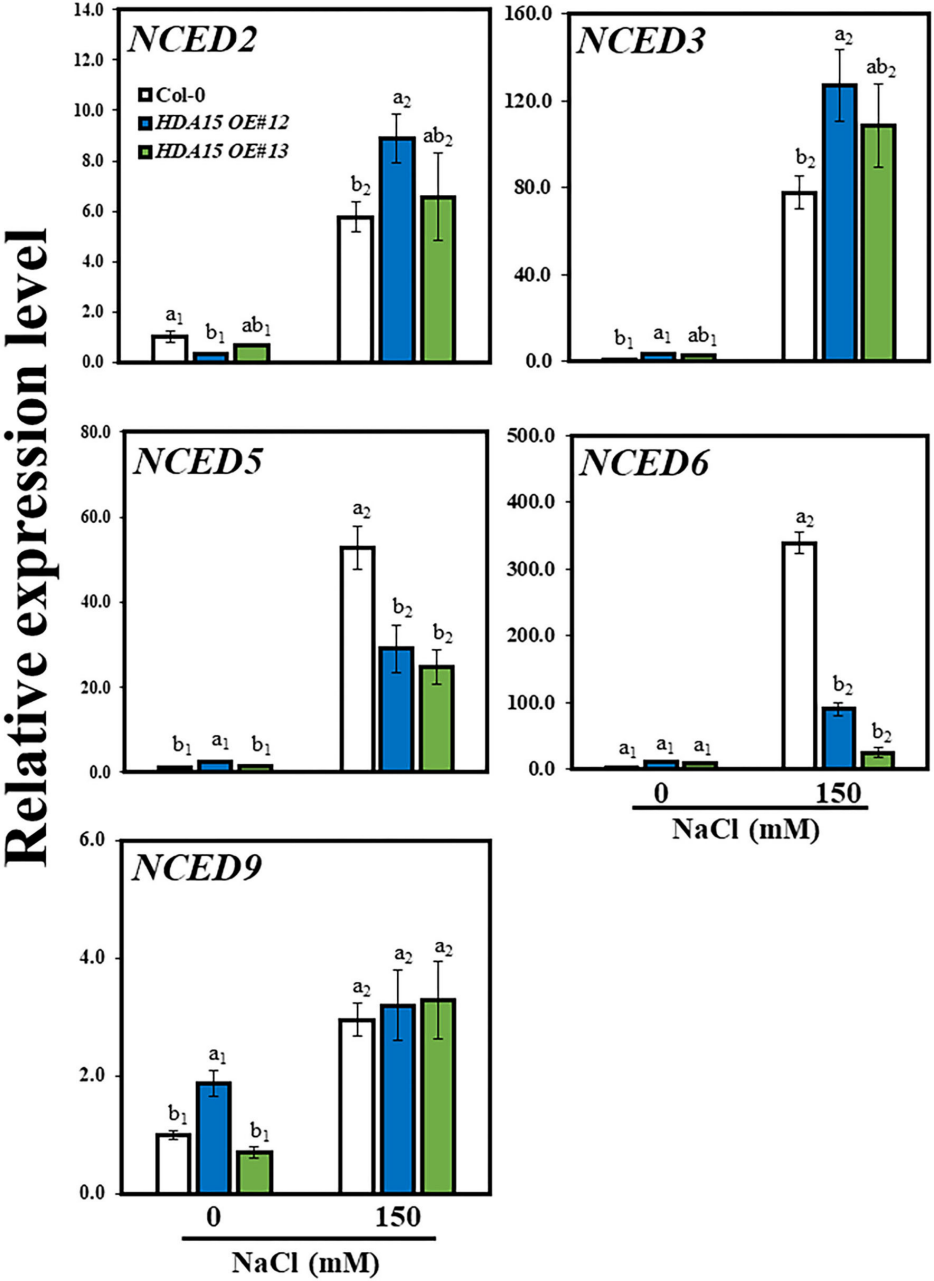
The expression levels *NCED* genes, which are abscisic acid (ABA) biosynthetic genes, under salt stress. Seven-day-old plants were subjected to 150 mM NaCl for 6 h. RNA was then extracted, and cDNA was synthesized. Next, qRT-PCR was performed with primers for *NCED2, NCED3, NCED5, NCED6*, and *NCED9* genes. *Actin2* was used as an internal control. The experiment was repeated three times independently. Error bars represent the standard deviation of three replicates. Different letters (a, b) indicate significant differences based on one-way ANOVA (*P* < 0.05).

**Figure 6:**
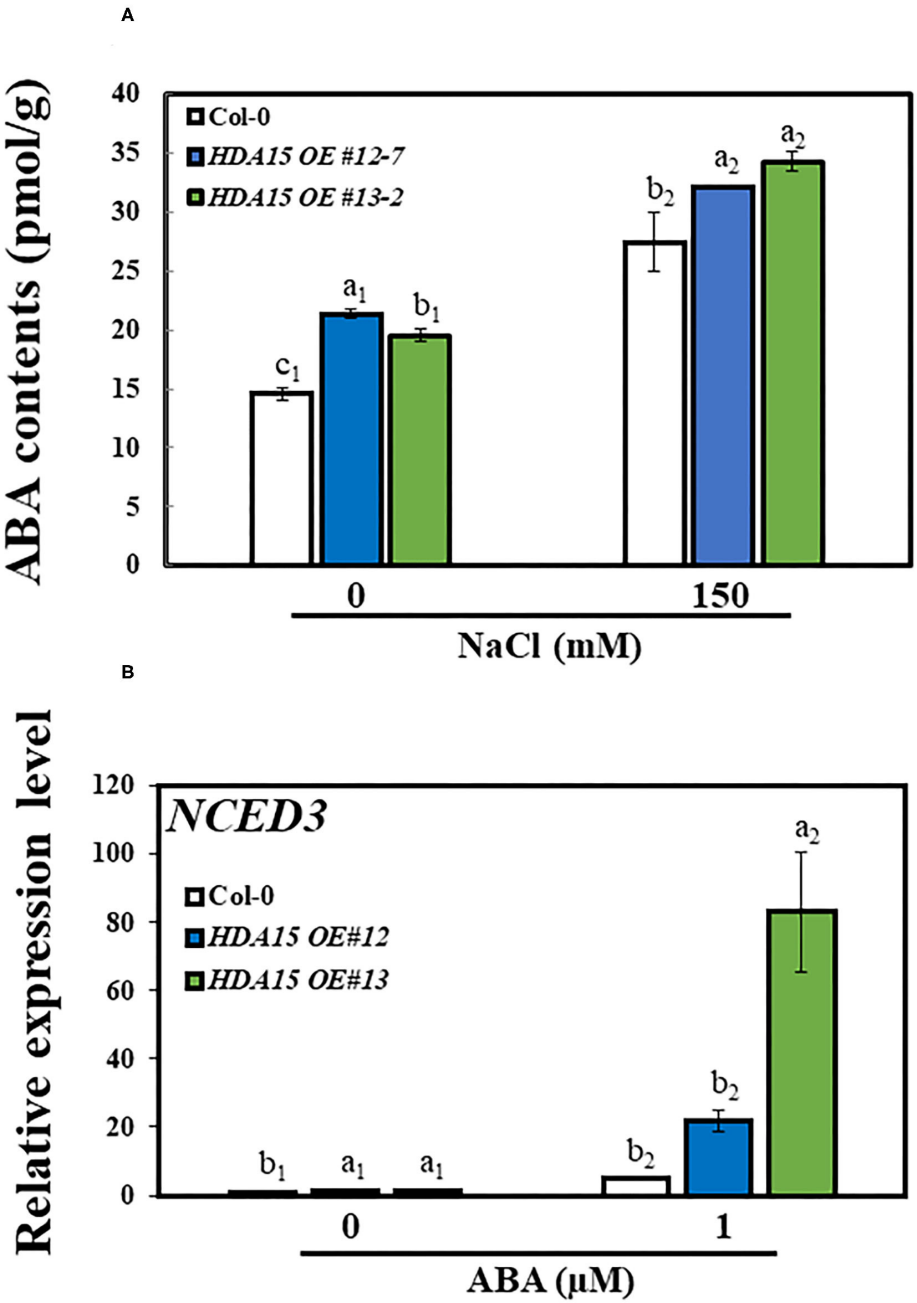
The accumulation of ABA in response to salt stress. **(A)** ABA contents. Seven-day-old plants were exposed to salt stress for 24 h, following which the contents of ABA were measured in Col-0 and *HDA15 OE* plants. **(B)** The expression level of *NCED3* in response to ABA treatment in Col-0 and *HDA15 OE* plants. Seven-day-old plants were challenged with 1 μM ABA for the measurement of transcript levels in Col-0 and *HDA15 OE* plants. Error bars represent the standard deviation of three replicates. Different letters (a, b, or c) within a treatment group indicate significant differences based on one-way ANOVA (*P* < 0.05).

A previous study suggested that HDA15 plays an important role in ABA signaling (Lee and Seo, 2019). Therefore, we checked the response of ABA biosynthetic genes *NCED2, NCED3, NCED5, NCED6*, and *NCED9* (Figure 5). The results indicated that the transcript levels of three *NCED* genes, *NCED2, NCED3*, and *NCED9*, were enhanced under salt stress. In detail, the transcript levels of *NCED2* and *NCED9* were marginally increased under salt stress in *HDA15 OE* mutants compared to that of Col-0 plants. However, *NCED3* transcript levels were ~1.5 times higher in two transgenic *HDA15* plants than in Col-0 plants under salt stress treatment. The expression levels of both *NCED5* and *NCED6* in *HDA15* OE plants were significantly downregulated in response to salt stress compared to those of Col-0 plants. *NCED3* is upregulated by salt and drought (Barrero et al., 2006). It appears that suppression of *NCED5* and *NCED6* leads to the upregulation of *NCED3*. Therefore, we postulated that *NCED3* may act as an important gene that affects ABA biosynthesis in response to salt stress in *HDA15* OE plants. This result also indicated that ABA enhances salt stress tolerance of *HDA15 OE* plants, which led us to determine the ABA content in Col-0 and *HDA15 OE* plants under salt stress (Figure 6A). *HDA15 OE* plants showed a higher ABA content than Col-0 plants when challenged by salt stress. Consistent with these results, we observed that *NCED3* expression was induced in OE plants exposed to ABA (Figure 6B). In addition, the transcript levels of *HDA15* were increased in response to ABA (Supplementary Figure 4).

**Figure 7:**
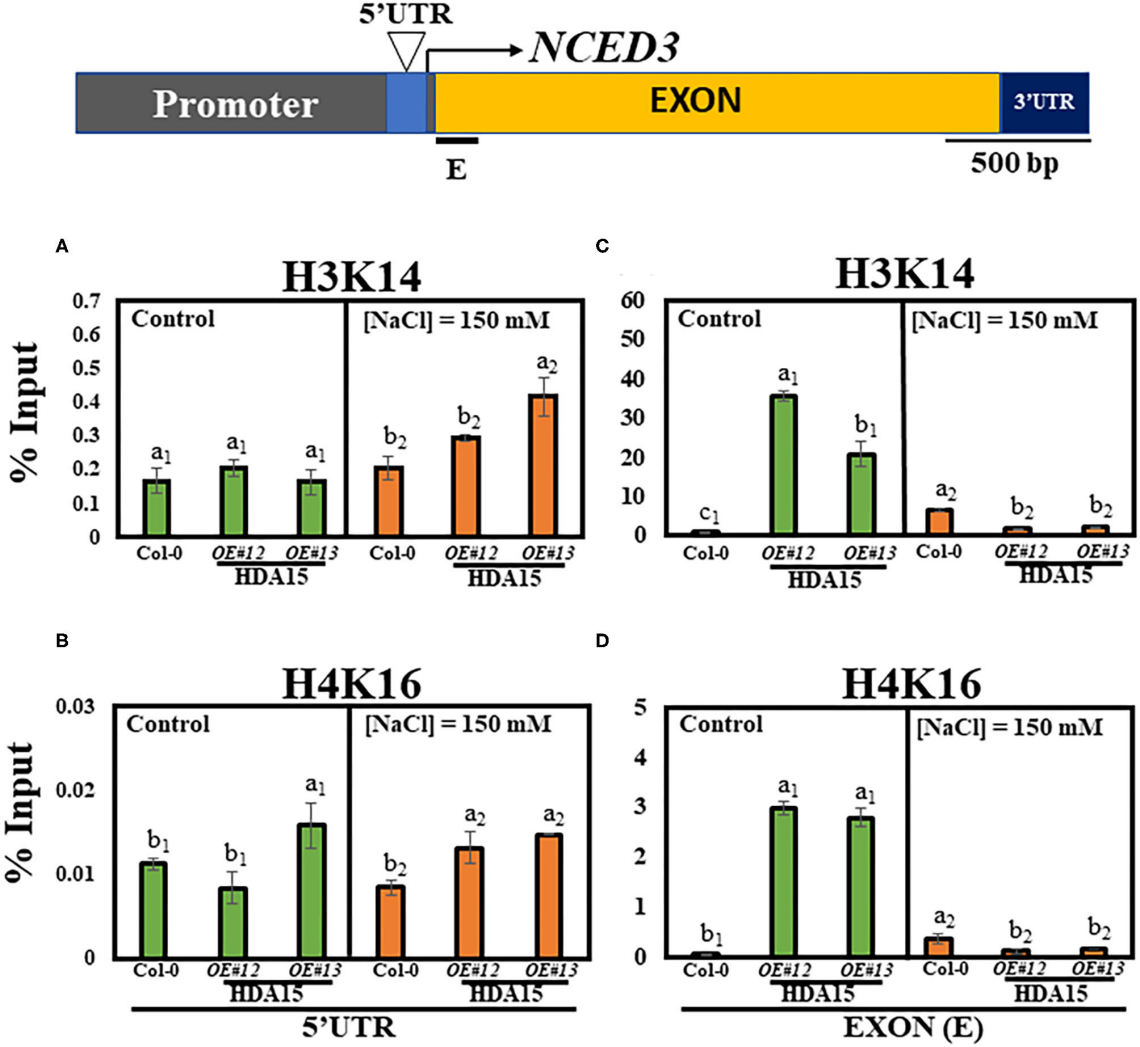
H3K14 and H4K16 levels in Col-0 and *HDA15 OE* transgenic plants in response to salt stress. Two-week-old plants were subjected to 150 mM salt stress for 6 h. The samples were then used for chromatin immunoprecipitation (ChIP) assay. Immunoprecipitated DNA was analyzed *via* qRT-PCR. **(A)** H3K14 levels at 5′UTR of *NCED3* promoter under normal and salt stress conditions. **(B)** H4K16 levels at 5′UTR of *NCED3* promoter under normal and salt stress conditions. **(C)** H3K14 levels at *NCED3* exon under normal and salt stress conditions. **(D)** H4K16 levels at *NCED3* exon under normal and salt stress conditions. Error bars represent the standard deviation of three replicates. Different letters (a, b, or c) within a treatment group indicate significant differences based on one-way ANOVA (*P* < 0.05).

Since HDA15 mediates histone deacetylation, which usually inhibits the expression of its target genes, we conducted a histone chromatin immunoprecipitation (ChIP) assay to determine the acetylation levels of *NCED3* in 5′UTR and exon regions. H3K14 and H4K16 levels at the 5′UTR were similar between control and salt stress conditions (Figures 7A,B). The NCED3 activator protein, NGATHA1, binds to the 5′UTR region of the *NCED3* promoter under drought conditions (Sato et al., 2018). It appears that HDA15 does not interact with NCED3 activator to regulate the deacetylation levels at this 5′UTR region. However, a significant decrease in H3K14 and H4K16 levels was observed at the first exon region of the *NCED3* (Figures 7C,D) in *HDA15 OE* plants under high salt stress. Since HDA15 acts as a negative regulator of certain genes, it is plausible that HDA15 increases deacetylation levels at the *NCED3* exon region, leading to the inhibition of binding by unknown negative regulators.

### Abscisic Acid Contents in *HDA15 OE* Plants Are Tightly Modulated *via* Abscisic Acid Homeostasis During Stress Response

**Figure 8:**
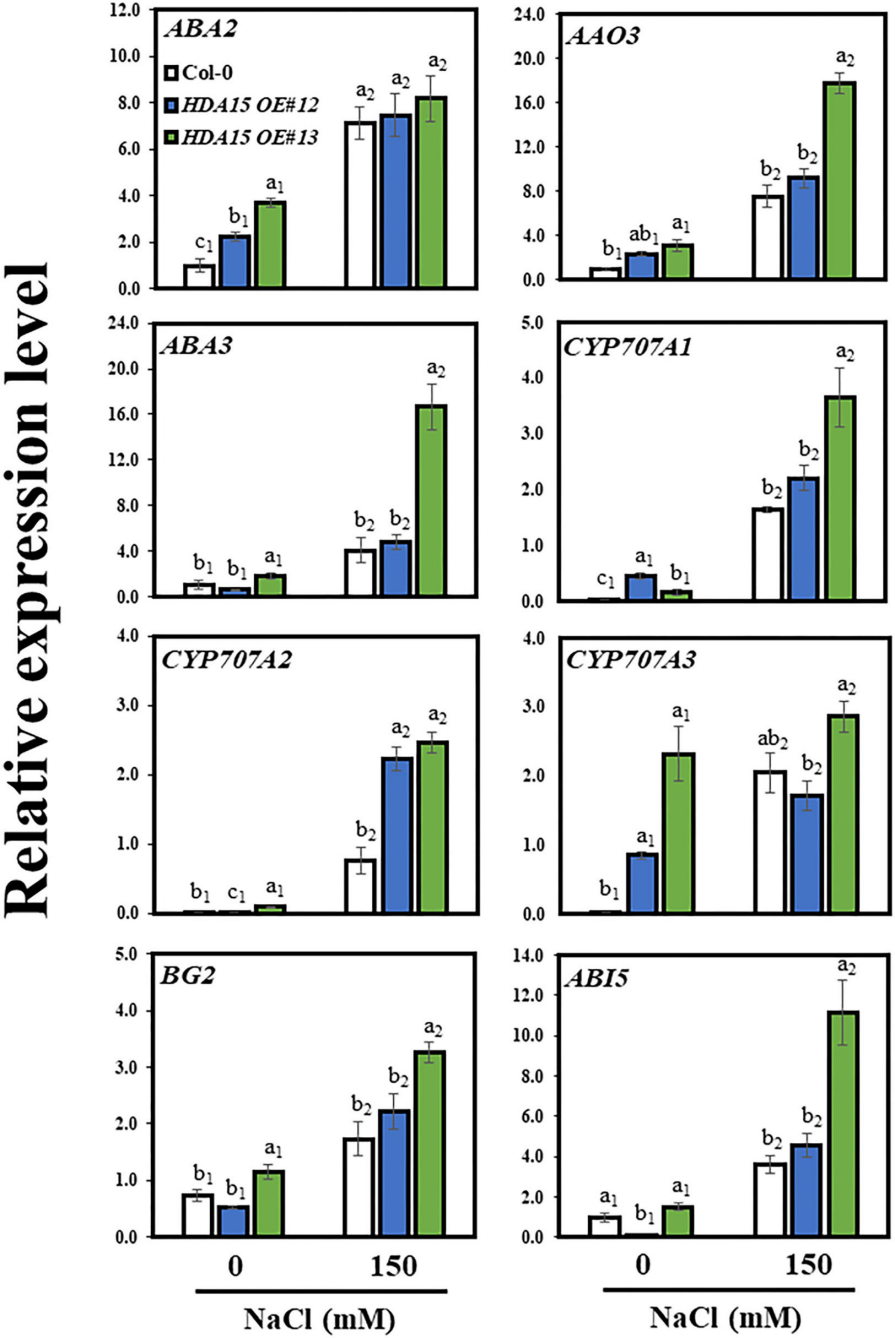
Expression of genes downstream of *NCED3* in abscisic acid (ABA) biosynthetic and catabolic pathways. Seven-day-old plants were subjected to 150 mM NaCl for 6 h. RNA was then extracted, and cDNA was synthesized. Primers for *ABA2, ABA3, AAO3, BG2, CYP707A1, CYP707A2, CYP707A3, CYP707A4*, and *ABI5* were used to perform qRT-PCR. *Actin2* was used as an internal control. The experiment was repeated three times independently. Error bars represent the standard deviation of three replicates. Different letters (a, b, or c) were considered significant differences based on one-way ANOVA (*P* < 0.05).

Because *NCED3* upregulation is partly responsible for ABA accumulation in *HDA15 OE* plants, we investigated the regulation of genes downstream of *NCED3* under salt stress. We assessed the transcript levels of *ABA2, ABA3, AAO3*, and *CYP707As* (Figure 8). The transcript levels of *ABA2, ABA3*, and *AAO3* in *HDA15 OE* plants were slightly higher than those of Col-0 under salt stress conditions. NCED3 reportedly serves a crucial role in ABA biosynthesis *via* its involvement in ABA biosynthetic pathways (Ma et al., 2018). In addition, two ABA catabolic genes, *cytochrome P450* (*CYP*) *707A1* (*CYP707A1*) and *CYP707A2*, were upregulated in *HDA15 OE* plants in response to salt stress compared to those in Col-0 plants. However, there was no difference between the *CYP707A3* transcript levels of Col-0 and *HDA15 OE* plants under stress conditions. Although ABA enables plants to adapt to abiotic stresses, excess internal ABA may negatively regulate plant growth (Belin et al., 2009; Yao and Finlayson, 2015). Thus, the increased expression of *CYP707As* may be a result of efforts to regulate ABA homeostasis in order to maintain cellular ABA levels that are optimal for plant growth. Furthermore, we determined that the expression of *BG2*, which hydrolyzes ABA-GE to ABA, induced by salt stress in *HDA15 OE* plants is higher compared to that in Col-0 plants. This also increases ABA accumulation in *HDA15 OE* plants under salt stress. We also examined the transcript levels of *ABI* genes. The transcript level of *ABI5* was higher in *HDA15 OE* plants than that in Col-0 plants. During seedling development, ABI5 induces certain *LEA* genes, thereby enhancing adaptation of plants against abiotic stresses (Skubacz et al., 2016). Additionally, levels of *ABI3* and *ABI4* in *HDA15 OE* plants were upregulated in response to salt stress (Supplementary Figure 5). ABI3, ABI4, and ABI5 proteins reportedly act together during stress responses (Skubacz et al., 2016). Although ABI1 and ABI2 are negative regulators of the ABA signaling pathway, their transcript levels, especially that of *ABI2*, were also enhanced by salt stress in *HDA15 OE* plants. These results may be due to increased ABA content, which activates the negative feedback loop in the ABA signaling pathway (Merlot et al., 2001).

### Interaction Between HY5 and HDA15 Is Crucial for Salt Stress Tolerance

**Figure 9:**
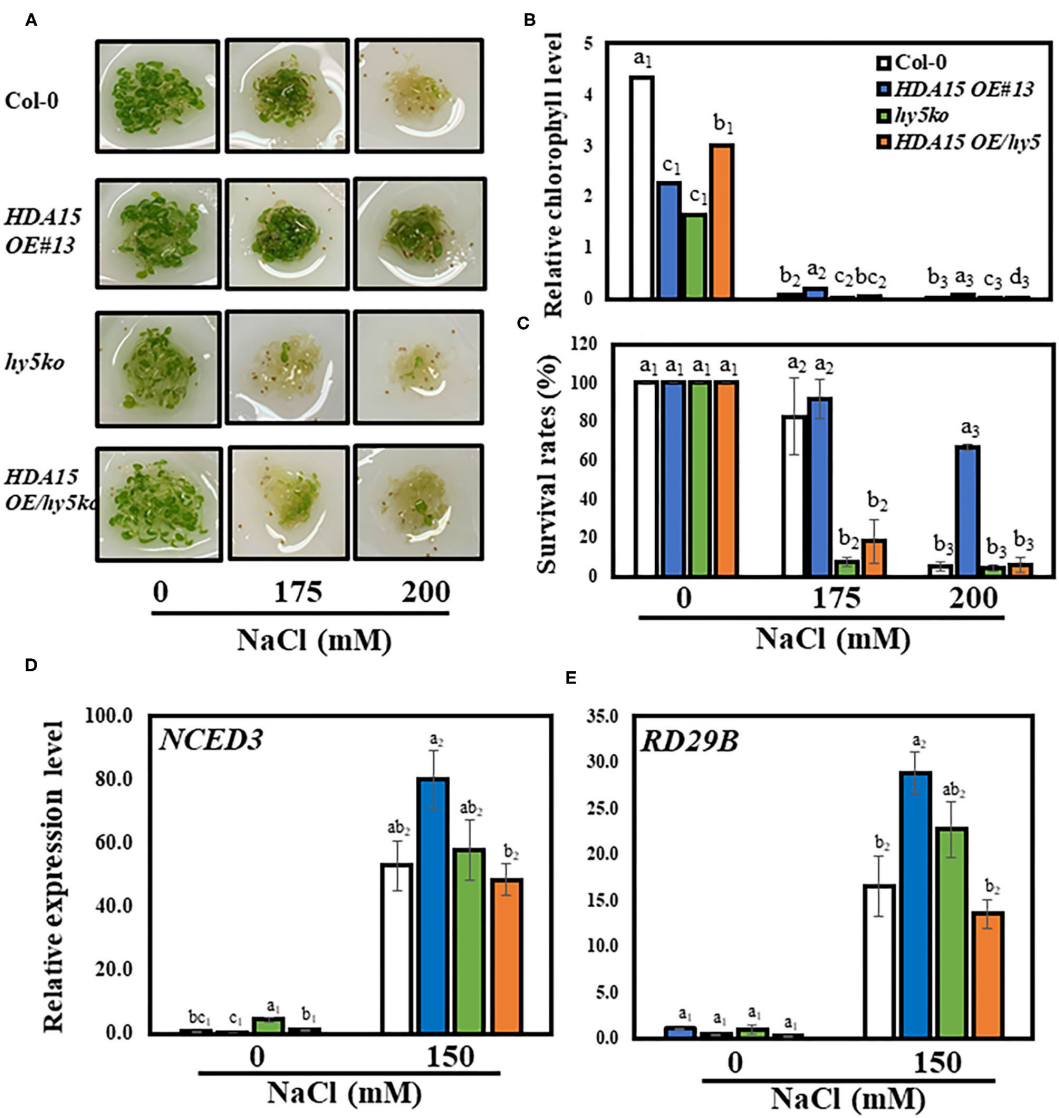
The co-action of HDA15 and HY5 in response to salt stress. Three-day-old plants germinated in normal MS media were transferred to different NaCl concentrations as indicated. The experiment was repeated three times independently to obtain a consistent result. **(A)** The phenotype of Col-0, *HDA15 OE/hy5 ko, hy5 ko* mutants, and *HDA15 OE* plants following a 4 d exposure to salt stress. **(B)** Chlorophyll levels of tested plants following a 4 d exposure to salt stress. **(C)** The expression levels of *HY5* in Col-0 and *HDA15 OE* plants. **(D,E)** The expression levels of *NCED3* and *RD29B* in Col-0, double mutants, *HDA15 OE*, and *hy5* ko plants. Seven-day-old plants were exposed to 150 mM NaCl for 6 h followed by RNA extraction and cDNA synthesis for qRT-PCR. *Actin2* was used as an internal control. Error bars represent the standard deviation of three replicates. Different letters (a, b, or c) within a treatment group indicate significant differences based on one-way ANOVA (*P* < 0.05).

The TF, HY5, regulates the association between HDA15 protein and its target genes (Zhao et al., 2019). Thus, we speculated whether HY5 also plays a role in enhanced salt stress tolerance conferred by *HDA15* overexpression. To address this issue, we generated a double-mutant by crossing *hy5* mutant with *HDA15 OE* plants (Supplementary Table 1) and tested its tolerance in response to salt stress. All tested plants exhibited the normal phenotype under control conditions (Figures 9A–C). However, when grown in salt medium containing 175 or 200 mM NaCl, the *HDA15 OE/hy5 ko* plants were more sensitive to salt stress compared to other tested plants. *HDA15 OE* plants are the most tolerant to salt stress. The results of chlorophyll content determination also substantiated the phenotype test (Figure 9B). Thus, our observation indicated that HDA15 may also interact with HY5 to confer salt stress tolerance to plants. Moreover, our assessment of *HY5* expression in *HDA15 OE* plants under salt stress indicated that HY5 transcript levels in *HDA15 OE* plants were upregulated (Supplementary Table 1). We also determined the transcript levels of *RD29B* and *NCED3* in response to salt stress in *HDA15 OE/hy5 ko* plants and found that both these genes were downregulated (Figures 9D,E).

## Discussion

Presently, most studies have demonstrated the function of *HDA15* in response to light (Liu et al., 2013; Gu et al., 2017). These indicate that both PIF1 and PIF3 interact with HDA15 in the dark to co-target and suppress a set of light-responsive chlorophyll biosynthetic and photosynthetic genes associated with hormone signaling (Liu et al., 2013; Gu et al., 2017). The role of *HDA* in response to salt stress has been revealed *via* studies on *HDA6, HDA9, HDA19, AtHD2A*, and *AtHD2D* (Sridha and Wu, 2006; Chen et al., 2010; Han et al., 2016; Zheng et al., 2016; Ueda et al., 2017). The most recent study indicated that *HDA15* plays a positive role in ABA signaling by suppressing negative regulators of ABA, which enables plants to tolerate drought stress (Lee and Seo, 2019). Reportedly, a quadruple mutant showing loss of function of all four class II HDACs (*HDA5/14/15/18*) displayed sensitivity to salt stress (Ueda et al., 2017). However, no further detailed research has been conducted on the function of HDA15 alone under salt stress. Therefore, in this study, we aimed to characterize the function of the *HDA15* in response to salt stress. We observed that the transcript level of *HDA15* was increased in response to salt stress. Additionally, overexpression of *HDA15* conferred salt stress tolerance to *Arabidopsis*, indicating the involvement of *HDA15* in salt stress tolerance in these plants. We also examined the phenotype of *hda15 ko* mutants in response to salt stress, but these were similar to that of Col-0. The reason for this may be that *HDA15* is one of several homologs belonging to class II of HDACs, which includes *HDA5, HDA14*, and *HDA18*. Only the quadruple mutant, *hda5/14/15/18*, showed a phenotype that was sensitive to salt stress (Ueda et al., 2017). Therefore, the loss of function of *HDA15* alone may not exert a phenotypic effect in response to salt stress due to the compensatory role of other homologs. Furthermore, since *HDA15 OE* transgenic plants showed tolerant phenotypes against salt stress, we utilized Col-0 and *HDA15 OE* plants to characterize the function of *HDA15*. Previous studies also proposed that overexpression of *AtHD2A* and *AtHD2D* in *Arabidopsis* increased tolerance to salt stress (Sridha and Wu, 2006; Chen et al., 2010; Han et al., 2016; Zheng et al., 2016, 2019; Ueda et al., 2017).

As HDA15 is involved in ABA signaling and ABA accumulation enhances salt stress tolerance (Sah et al., 2016; Lee and Seo, 2019), we examined the effect of salt stress on the transcript levels of ABA biosynthetic genes. NCEDs are enzymes that mediate ABA biosynthesis. To date, five NCED genes are known to be present in *Arabidopsis*. These include AtNCED2, AtNCED3, AtNCED5, AtNCED6, and AtNCED9 (Ali et al., 2020). *NCED3*, which is induced by both ABA and NaCl, plays a vital role in osmotic stress-induced ABA biosynthesis (Iuchi et al., 2000; Tan et al., 2003; Barrero et al., 2006). Moreover, overexpression of *OsNCED3* in rice conferred protection against osmotic stress (Huang et al., 2018). *NCED3* induction increased ABA biosynthesis, resulting in enhanced ABA accumulation, which speeds up stomatal closure and upregulates the expression of stress-responsive genes, leading to increased stress tolerance in plants (Jakab et al., 2005). Drought-related stress causes the histone methyltransferase, ATX1, to modify H3K4me3, which then activates NCED3, resulting in drought- and ABA-related genes being upregulated (Kim et al., 2015). As shown in *Arabidopsis*, overexpression of GmWRKY16, a soybean WRKY TF, increases ABA accumulation, which is also observed when *NCED3* is upregulated, enabling transgenic plants to resist drought and salt stresses (Ma et al., 2019). Thus, upregulation of *NCED3* in *HDA15 OE* plants may be a key factor in osmotic stress tolerance, with particular reference to salt stress. The increased transcript levels of *NCED3* led us to examine other downstream genes involved in the ABA biosynthetic pathway. *NCED* genes catalyze the synthesis of xanthoxin, which is converted to abscisic aldehyde by short-chain alcohol dehydrogenase/reductase (SDR/ABA2) and then to ABA by abscisic aldehyde oxidase (AAO3). The molybdenum cofactor sulfurase/ABA3 is required by aldehyde oxidase for its activity (Long et al., 2019). However, as ABA2, ABA3, and AAO3 play only minor roles in ABA biosynthetic pathway (Ma et al., 2018), their transcript levels were not significantly increased in *HDA15 OE* plants (Figure 8). In addition, the higher accumulation of ABA in *HDA15 OE* plants may be due to *BG2* upregulation, which catalyzes inactive ABA-GE to ABA in one-step hydrolysis (Long et al., 2019). Induction of *BG2* also helps plants to quickly enhance internal ABA levels for stress adaptation. On the other hand, cytochrome P450 monooxygenase 707A family members (CYP707As) are responsible for the degradation of ABA levels in plants. The mRNA levels of CYP707As are modulated by abiotic stresses (Yoon et al., 2020). Our results showed that the transcript levels of *CYP707A1* and *A2* in *HDA15 OE* plants were enhanced by salt stress. Such induction may help plants to prepare for ABA degradation when stress is removed. Although ABA is a key hormone in abiotic stress adaptation, where increased ABA content enables plants to tolerate unfavorable environments, excess ABA levels exert adverse effects on plant growth (Belin et al., 2009; Yao and Finlayson, 2015). Therefore, ABA homeostasis is important to balance plant growth and stress response in order to obtain the best growth performance under a given condition. In plants, the processes involved in the biosynthesis and catabolism of ABA must balance each other to maintain ABA contents at optimal levels during osmotic stress. Thus, *CYP707A* upregulation may be intended to maintain ABA levels within the optimal range in plants subjected to stress (Liu S. et al., 2014; Long et al., 2019). Therefore, while increasing internal ABA contents to cope with salt stress, *HDA15 OE* plants maintain ABA homeostasis by inducing the ABA catabolic compound, CYP707As, in order to balance and optimize ABA levels during the course of stress response and adaptation.

HDA15 displays deacetylase activity, which mediates the modulation of gene repression (Liu et al., 2013; Perrella et al., 2013). Many transcriptional repressors recruit HDA proteins to form a complex to regulate their target genes. Interaction between HDACs and other proteins creates a structural link between DNA, histones, and core deacetylase enzymes (Perrella et al., 2013). For instance, PIF3, a negative regulator of light signaling, reportedly interacts with HDA15 to suppress chlorophyll biosynthesis and photosynthesis in the dark (Liu et al., 2013). HDA15 and HY5 reportedly co-modulate hypocotyl elongation, cell wall, and auxin-related genes (Zhao et al., 2019). HY5 is a master transcription regulator not only of light response but also of abiotic stress (Chen et al., 2008; Gangappa and Botto, 2016; Yang et al., 2018). We found that the salt stress-tolerant phenotype of *HDA15 OE* was absent in *HDA15 OE/hy5 ko* plants, indicating that HY5 and HDA15 may form a complex that regulates salt stress response in *Arabidopsis*. In addition to HDA15, HDA19 can also be recruited by the AP2/EREBP TF, AtERF7, in response to ABA and drought stress to form a suppressor complex with AtSin3 to suppress the expression of stress-responsive genes (Song et al., 2005). HDA19 also regulates ABA synthesis by forming a repressive complex with SNL2 when interacting with SNL1 (Wang et al., 2013). Moreover, the transcript levels of ABI3 are modulated by the BES1-TPL-HDA19 suppressor complex *via* histone deacetylation (Ryu et al., 2014). *HDA19 RNAi* plants were found to be insensitive to salt stress, and the MSI1-HDA19-SIN3 complex modulated ABA receptor genes involved in ABA signaling (Mehdi et al., 2016). Considered together, our results demonstrated that although HDA15 protein is expected to inhibit the expression of specific genes by promoting histone deacetylation, it may actually exert the opposite effect (Figure 7). Therefore, further studies, such as those utilizing clustered regularly interspaced short palindromic repeats (CRISPR) technology, are required to identify regions within target DNA that HDA affects in order to regulate gene expression. Such studies may contribute to the development of salt-resistant crop varieties.

## Conclusions

In this study, we found that overexpression of *HDA15* in *Arabidopsis* increased the transcription levels of *NCED3*, which further enhanced ABA synthesis, resulting in resistance to salt stress. We also found that HDA15 requires HY5 to increase the resistance of plants to salt stress. Investigation of the mechanism by which HDA15 increases the transcript levels of *NCED3* in response to salt stress indicated that it possibly involves the inhibition of a negative regulator that binds this site *via* increased histone deacetylation of a specific region in the DNA of *NCED3*. However, further studies are needed to clearly identify negative regulators that are inhibited by HDA15 in order to fully understand its function under salt stress.

## Data Availability Statement

The original contributions presented in the study are included in the article/Supplementary Material, further inquiries can be directed to the corresponding authors.

## Author Contributions

HT conducted the phenotype experiment, ChIP assay, qRT-PCR analysis, statistical analysis, and wrote the manuscript. SL performed the GUS experiment and ABA content assays. CT carried out the chlorophyll assay. WL conducted the MDA assay. E-HC did the proline assay. S-WH and HL designed the experiments and wrote the manuscript. All authors contributed to the article and approved the submitted version.

## Conflict of Interest

The authors declare that the research was conducted in the absence of any commercial or financial relationships that could be construed as a potential conflict of interest.
