# Supplementary figures and images for: Overexpression of the HDA15 Gene Confers Resistance to Salt Stress by the Induction of NCED3, an ABA Biosynthesis Enzyme
Source: Front Plant Sci. 2021 Apr 30;12:640443. doi: 10.3389/fpls.2021.640443 (PMC8120240; doi:10.3389/fpls.2021.640443)

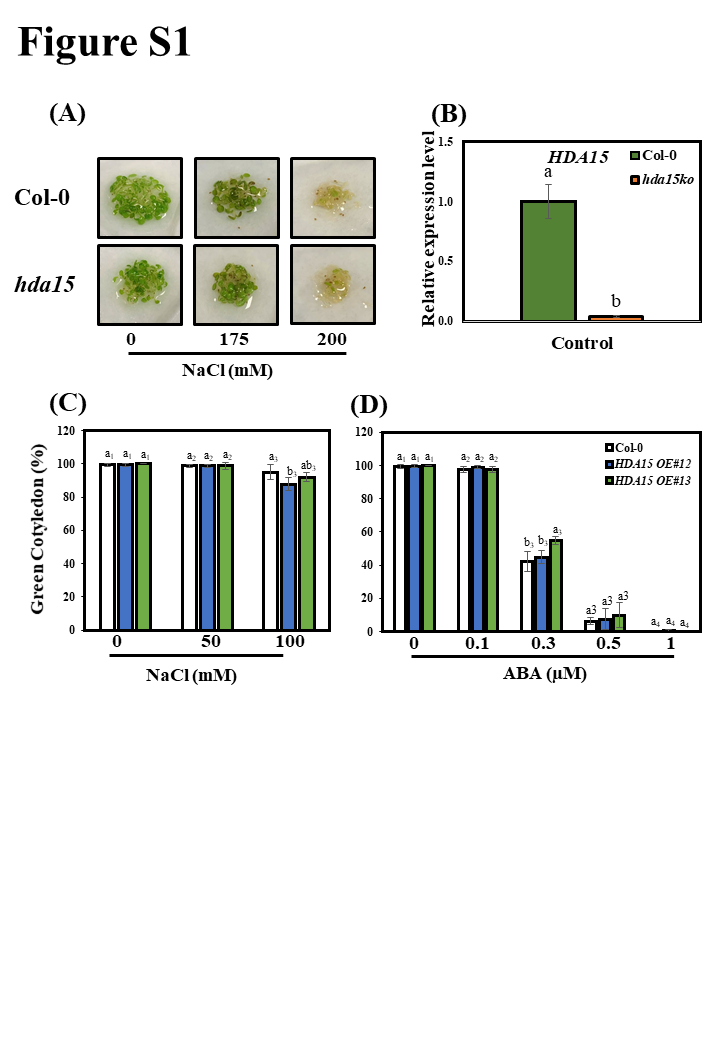

Supplement: Supplementary file 1 [file Image_1.TIF]

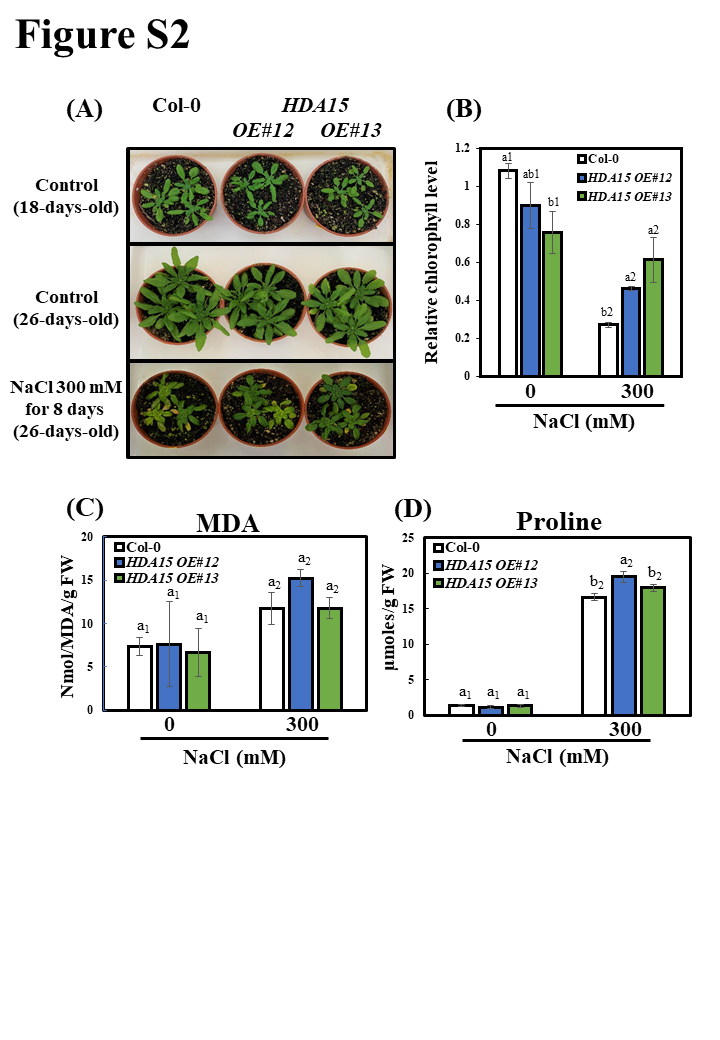

Supplement: Supplementary file 2 [file Image_2.TIF]

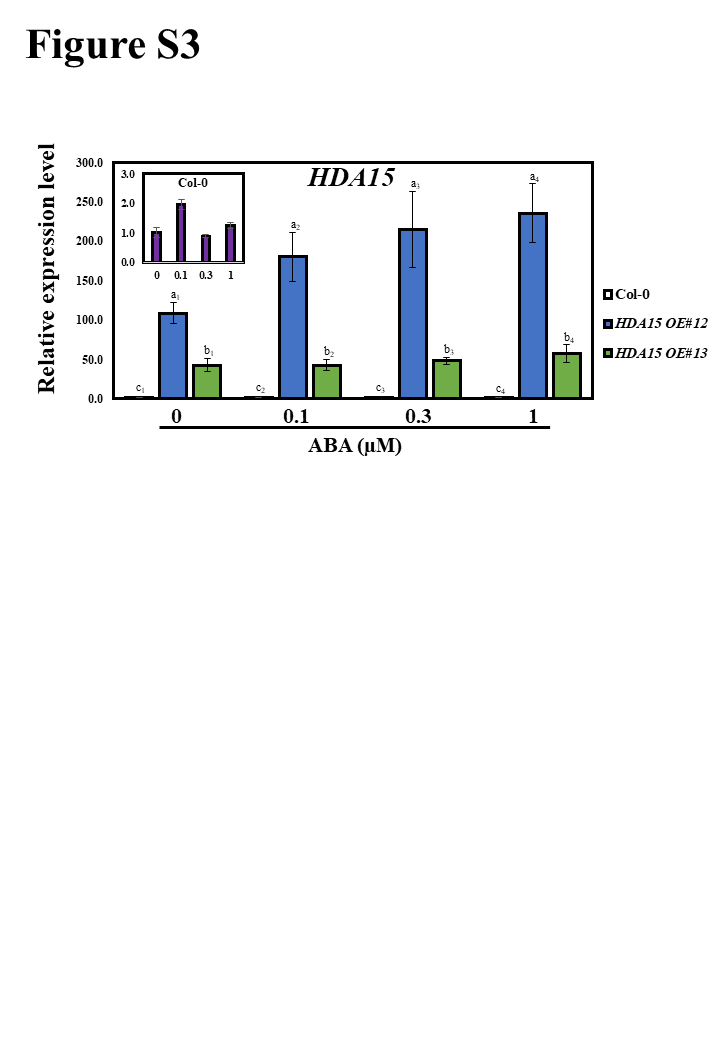

Supplement: Supplementary file 3 [file Image_3.TIF]

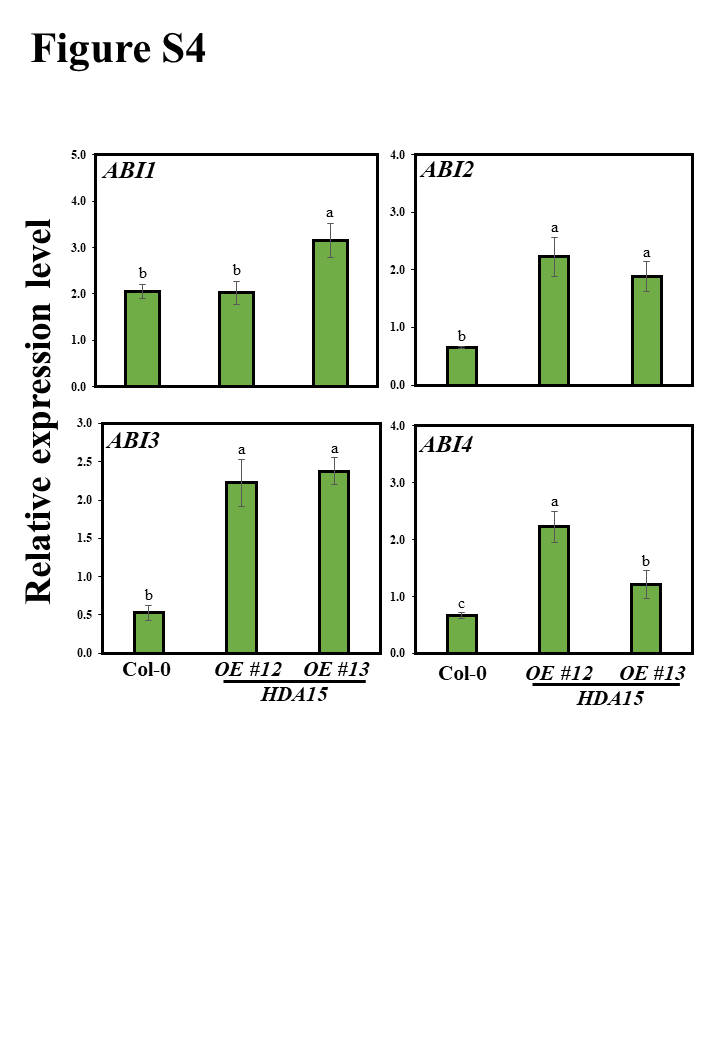

Supplement: Supplementary file 4 [file Image_4.TIF]

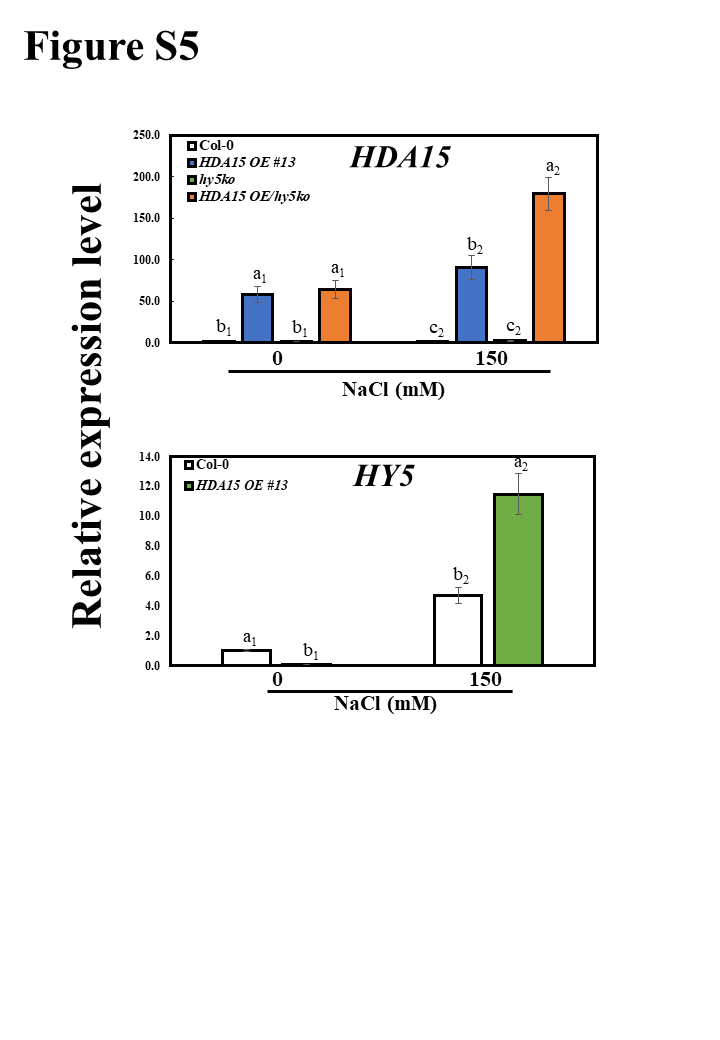

Supplement: Supplementary file 5 [file Image_5.TIF]
