# Supplementary material for: Overexpression of the HDA15 Gene Confers Resistance to Salt Stress by the Induction of NCED3, an ABA Biosynthesis Enzyme
Source: Front Plant Sci. 2021 Apr 30;12:640443. doi: 10.3389/fpls.2021.640443 (PMC8120240; doi:10.3389/fpls.2021.640443)

Supplementary Materials

# Supplementary Figures and Tables

## Supplementary Figures

**Fig S1**: **Growth performance of Col-0, *hda15ko* and *HDA15 OE* plants in response to salt stress**

Four-day-old plants, germinated in normal MS media, were transferred to test medium with different NaCl concentrations as indicated. The experiments were repeated thrice independently. (A) The phenotype of Col-0 and *hda15 ko* mutants after a 3 d exposure to salt stress. (B) *HDA15* expression in Col-0 and *hda15 ko* mutant plants. Seven day old plants were utilized for RNA extraction and cDNA synthesis for qRT-PCR. *Actin2* was used as an internal control. (C) The germination rates of Col-0 and *HDA15* *OE* plants in 0, 50, 100 mM NaCl. (D) The germination rates of Col-0 and *HDA15* *OE* plants in 0, 0.1, 0.3, 0.5 and 1 µM ABA. The results were obtained after 8 d from the day of germination and presented as percentages of green cotyledons. (E) The lipid peroxidation levels of Col-0 and *HDA15 OE* plants in response to salt stress. Seven day old plants, which were germinated in normal MS media, were transferred to 150 mM NaCl for 0, 3, and 6 h. Error bars represent the standard deviation of three replicates. Different letters (a, b, or c) within a treatment group indicate significant difference based on one-way ANOVA (P < 0.05).

**Fig S2**: **Growth performance of Col-0 and *HDA15 OE* plants grown in soils under salt stress**

(A) Four-week-old plants grown in soils were watered with 0 or 300 mM salt solution for 8 days. The photos were taken before and after being exposed to salt stress. (B) The chlorophyll contents of Col-0, *HDA15 OE* plants in response to salt stress at vegetative stage. (C) The lipid peroxidation levels of Col-0 and *HDA15 OE* plants in response to salt stress at vegetative stage. (D) The proline content of Col-0 and *HDA15 OE* plants in response to salt stress at vegetative stage. Error bars represent the standard deviation of three replicates. Different letters (a, b, or c) within a treatment group indicate significant difference based on one-way ANOVA (P < 0.05).

**Fig S3**: **The expression levels of *HDA15* in Col-0, *HDA15 OE* plants in response to exogenous ABA**

Seven-day-old plants, germinated in normal MS media, were transferred to 0, 0.1, 0.3, 0.5, and 1 µM ABA and incubated for 0 and 6 h. The treated plants were then utilized for RNA extraction and cDNA synthesis for qRT-PCR. *Actin2* was used as an internal control. Error bars represent the standard deviation of three replicates. Different letters (a, b, or c) within a treatment group indicate significant difference based on one-way ANOVA (P < 0.05).

**Fig S4**: **The expression levels of *ABI1*, *ABI2*, *ABI3*, *ABI4* in Col-0 and *HDA15 OE* plants under salt stress conditions**

Seven-day-old plants, germinated in normal MS media, were transferred to 150 mM NaCl and incubated for 6 h. The treated plants were then utilized for RNA extraction and cDNA synthesis for qRT-PCR. *Actin2* was used as an internal control. Error bars represent the standard deviation of three replicates. Different letters (a, b, or c) within a treatment group indicate significant difference based on one-way ANOVA (P < 0.05).

**Fig S5**: **The expression levels of *HDA15* in Col-0, *HDA15 OE, HDA15OE/hy5 ko* and *hy5 ko* plants and the expression levels of *HY5* in Col-0 and *HDA15 OE* plants in response to salt stress**

Seven day old plants, germinated in normal MS media, were transferred to 150 mM NaCl for 0 and 6 h. Treated plants were then utilized for RNA extraction and cDNA synthesis for qRT-PCR. *Actin2* was used as an internal control. Error bars represent the standard deviation of three replicates. Different letters (a, b, or c) within a treatment group indicate significant differences based on one-way ANOVA (P < 0.05).

## Tables

**Table S1**: The qRT-PCR primers used in this study


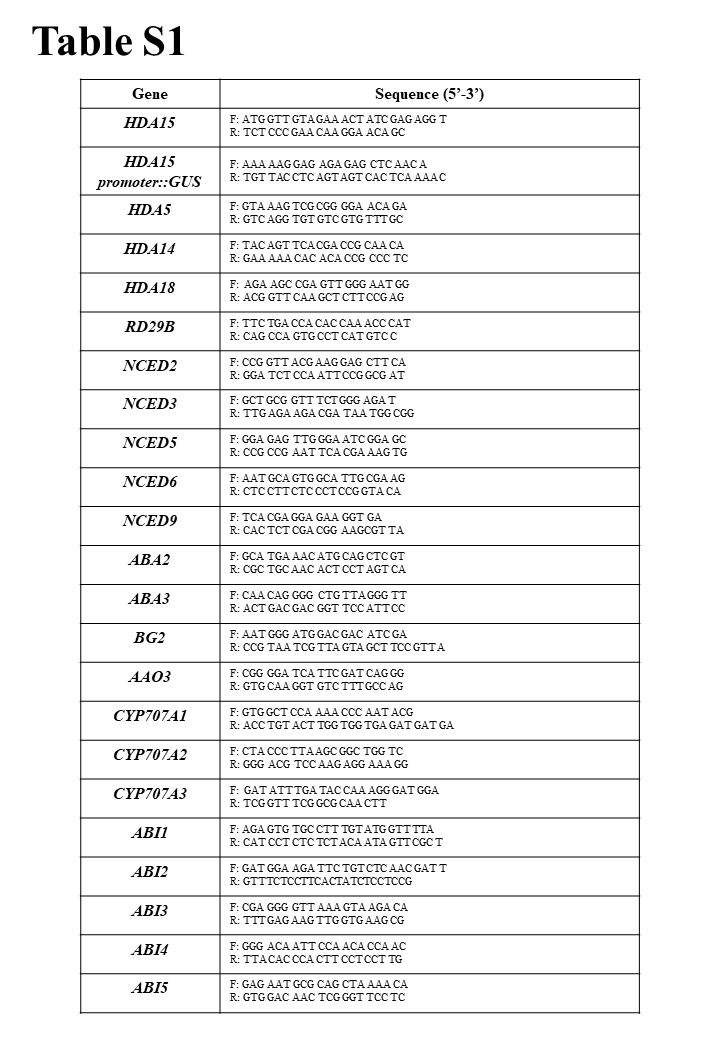

Supplement: Supplementary file 6 [file Data_Sheet_1.docx]
